# Incorporating Population Genomic Perspectives Into Kelp Conservation and Aquaculture in the Pacific Northwest

**DOI:** 10.1111/eva.70287

**Published:** 2026-07-01

**Authors:** Jordan B. Bemmels, Gregory L. Owens

**Affiliations:** ^1^ Department of Biology University of Victoria Victoria British Columbia Canada; ^2^ The Kelp Rescue Initiative Bamfield Marine Sciences Centre Bamfield British Columbia Canada

## Abstract

Recent kelp forest declines and growth in the kelp aquaculture industry have fueled increasing interest in ecological and evolutionary research on kelp forests, including kelp population genomics. Although many kelp management activities have inherent genetic and evolutionary implications, kelp management in the Pacific Northwest (PNW) of North America has to date made only limited use of species‐specific population genetic knowledge. We believe that kelp managers in the PNW are well positioned to begin routinely incorporating population genomic perspectives into their work. Here, we review the state of genetic knowledge in the canopy‐forming kelps *Nereocystis* and *Macrocystis* from Washington to Alaska and highlight how this knowledge can support four key kelp management activities: restoration, aquaculture, wild harvest, and biobanking. We discuss several potential paradigms for geographic transfer of genetic material, consider the likely impacts of inbreeding and genetic drift in the management of small kelp populations, and suggest strategies for protecting the genetic integrity of populations during wild harvest. To inform optimal sourcing strategies for biobanking and outplanting, we also reanalyze genomic data to explore how the number of individuals sampled impacts retention of genetic diversity. In many cases, predictions derived from molecular data and models have accumulated faster than the research community's ability to validate them in the field. We propose that experimental tests be incorporated into ongoing kelp management operations as an immediate step in transitioning toward a management framework informed by population genomic data and perspectives. Adopting such a framework will improve the likelihood of attaining desirable outcomes in kelp conservation and aquaculture, including as kelp populations adapt to future challenges.

## 1 Introduction

Kelp forests are seaweed‐dominated coastal marine ecosystems found throughout temperate and Arctic regions worldwide (Wernberg et al. 2019). They exhibit high net primary productivity (Mann 1973), provide habitat for biodiverse communities (Teagle et al. 2017), cycle carbon (Krause‐Jensen and Duarte 2016), and remove excess nutrients from ecosystems (Xu et al. 2023), providing an estimated ~$500 billion worth of ecosystem services annually (Eger et al. 2023). Kelps are also of longstanding cultural significance to coastal peoples (Dillehay et al. 2008; Turner 2001), are directly harvested from the wild (Mac Monagail et al. 2017), and are farmed in the global aquaculture industry (Grebe et al. 2019; Hu et al. 2021). The ecological, economic, and cultural importance of kelp contextualizes concern about recent kelp forest declines in many—though not all (Krumhansl et al. 2016)—regions of the world (Wernberg et al. 2019). Such declines have been attributed to factors including warming ocean temperatures (Smale 2020), marine heatwaves (Rogers‐Bennett and Catton 2019), loss of keystone predators that limit herbivore abundance (Estes and Duggins 1995; Galloway et al. 2023), and pollution from urban areas (Coleman et al. 2008). These declines have fueled increasing interest in restoring degraded kelp forests (Eger et al. 2022), improving monitoring and regulations concerning the commercial harvest of wild kelp (Carranza et al. 2024), and establishing germplasm biobanks to support conservation and aquaculture (Schenk et al. 2026; Visch et al. 2019; Wade et al. 2020).

Each of these activities—restoration, aquaculture, wild harvest, and biobanking—should ideally be informed by population genetic knowledge to maximize the potential for achieving desirable outcomes. Kelp restoration commonly involves the introduction of new individuals to a restoration area through transplanting sporophytes or seeding substrate with spores or gametophytes (Eger et al. 2022; Morris et al. 2020). Although ecological restoration projects across diverse taxa are frequently conducted without genetic knowledge (Mijangos et al. 2015), any deliberate movement of individuals from one location to another inherently involves genetic decisions such as the number of parent individuals represented and their geographic provenance. Aquaculture involves similar decisions, with implications not only for farmed populations but also for wild populations with which farmed individuals may interact (Hu et al. 2021). Failure to consider these implicit genetic decisions could result in poorly adapted or inbred kelp populations, populations with little capacity to adapt to future challenges, and unintended or potentially harmful genetic change in nearby local populations. Meanwhile, wild harvest that removes individuals inherently alters the size, composition, and connectivity of populations (Allendorf et al. 2008) and thus has the ability to alter local and regional gene pools and the evolutionary trajectories of wild populations. Finally, biobanks benefit from population genetic knowledge as they are explicitly envisioned as repositories for genetic material that can support conservation, restoration, and aquaculture (Peres 2016).

In some areas of the world, genetic considerations are already well incorporated into kelp management and cultivation. Breeding programs and genetic cultivar selection have supported kelp aquaculture in East Asia since at least the 1950s (Hu et al. 2024; Hwang et al. 2019). In South America, characterization of genetic structure and experimental tests of local adaptation and genetic crosses have provided detailed insights that could guide restoration and domestication of *Macrocystis* (Camus et al. 2018; Solas et al. 2024; Westermeier et al. 2010). In Australia, genetic structure has been used to select donor populations for restoration of extirpated *Phyllospora* populations (Wood et al. 2020), and warm‐tolerant genotypes have been identified and used in *Macrocystis* restoration trials (Layton and Johnson 2021). Despite such examples, in many regions of the world, the integration of genetic knowledge into active kelp management is still in its infancy. This situation may partly reflect a lack of basic information about genetic variability among wild kelp populations (Hu et al. 2023) and a lack of widespread understanding of the benefits of genetic knowledge and risks of ignoring it. Indeed, conservation practitioners frequently face barriers in expertise, funding, and collaboration that prevent them from incorporating genetic considerations into their work (Taylor et al. 2017). Conversely, academic conservation geneticists often fail to effectively communicate the practical implications of advanced genomic analyses to practitioners (Hogg 2024; Shafer et al. 2015) or to make any specific recommendations at all (Britt et al. 2018).

With these challenges in mind, we synthesize recent advances in population genetic knowledge of canopy‐forming kelps from the Pacific Northwest (PNW) of North America—here defined as the coasts of Washington (USA), British Columbia (BC, Canada), and southern Alaska (USA)—with the goal of translating complex genetic studies into a discussion of management options that is accessible to non‐specialists. We also reanalyze published genomic data to address simple but pertinent questions that have not been explored in existing literature. Although we focus on kelp in the PNW, many of the same general principles could be applied to management of kelp, other marine plants, or other species more generally and elsewhere in the world. We focus on population genetics, that is, the study of genetic differences among populations due to evolutionary forces such as migration, natural selection, genetic drift, and mutation (Hamilton 2021). We focus on the four management activities highlighted above—restoration, aquaculture, wild harvest, and biobanking—as we believe these are the areas with the greatest potential to benefit from population genetic knowledge. Overall, we argue that knowledge has advanced to the point where kelp stakeholders in the PNW are well poised to routinely incorporate population genetic perspectives into their work—though validating predictions derived from molecular data using lab or field experiments will often be a critical first step.

## 2 State of Population Genetic Knowledge in PNW Kelps

Throughout the PNW, bull kelp (*Nereocystis luetkeana*) and giant kelp (*Macrocystis tenuifolia*) are the main kelp species that create surface‐canopy forming kelp forests. Until recently (Lindstrom 2023), *M. tenuifolia* was considered part of a globally distributed monotypic species *M. pyrifera* (Demes et al. 2009) composed of four ecotypes with different holdfast morphologies, with both *integrifolia* and *pyrifera* morphs present in different parts of the PNW (S.C. Lindstrom, personal communication; Gonzalez et al. 2023; Macaya and Zuccarello 2010a; Saunders and McDevit 2014). Holdfast morphology in Californian *Macrocystis* is genetically determined (Gonzalez and Raimondi 2024), though the amount of genetic divergence between morphs is substantially lower in California (where the morphs partially overlap in distribution) than in the Southern Hemisphere (where morph distributions do not directly overlap) (Bemmels et al. 2026; Gonzalez et al. 2023). Given these complications and the need for further taxonomic work (Lindstrom 2023), we hereafter refer to all giant kelp as simply *Macrocystis*.

**FIGURE 1:**
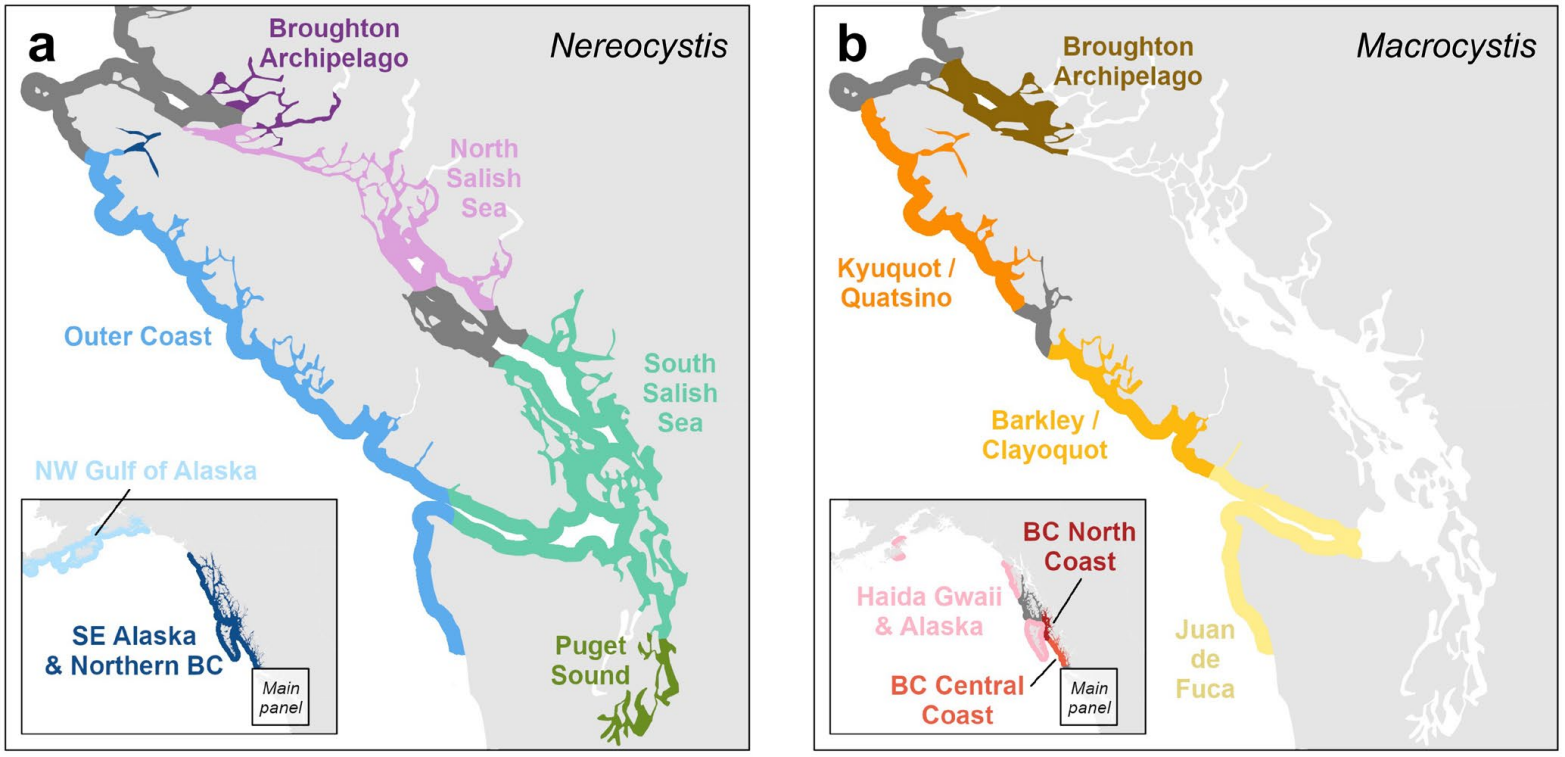
Genetic clusters based on presumably neutral genetic variants in southern British Columbia (BC) and Washington (main panels) and northern BC and Alaska (inset panels) for (a) *Nereocystis* and (b) *Macrocystis*. Areas of the coast corresponding to each genetic cluster are outlined in different colors, with dark grey outline indicating that no genetic samples are available from these regions and the predominant genetic cluster cannot be inferred. Genetic cluster distributions are simplifications redrawn from Bemmels et al. (2026) and all boundaries between genetic clusters are approximate. Simplified presence (coastal outline) or absence (no outline) of each species is based on data from ShoreZone (shorezone.org).

In both *Nereocystis* and *Macrocystis*, genetic studies have characterized geographic patterns of genetic diversity and genetic differences among populations. Studies using various genetic markers representing small subsets of the genome have been conducted range‐wide in *Nereocystis* (Gierke et al. 2023), and in *Macrocystis* within California (Alberto et al. 2010, 2011; Johansson et al. 2015), in the Southern Hemisphere (Camus et al. 2018; Iha, Layton, Amancio, et al. 2023; Iha, Layton, Flentje, et al. 2023; Le et al. 2024; Macaya and Zuccarello 2010b; Salavarría et al. 2018), and across hemispheres (Assis et al. 2023; Coyer et al. 2001; Gonzalez et al. 2023; Macaya and Zuccarello 2010a). More recently, whole genome sequences have revealed genetic patterns within the PNW in finer detail (Bemmels et al. 2025, 2026), identifying seven genetic clusters in each species (Figure 1). Genetic differentiation among populations is substantial, suggesting limited genetic connectivity (Bemmels et al. 2025, 2026). However, there are no ancient genetic splits known within the PNW as populations likely diverged during the Holocene or Last Glacial Period (Bemmels et al. 2026). In both species, genetic diversity is highest in California (Assis et al. 2023; Bemmels et al. 2025, 2026; Gierke et al. 2023), likely due to long‐term persistence of large, stable populations in California. Within the PNW, genetic diversity is highest in Haida Gwaii and southeast Alaska in *Nereocystis* and the Central Coast of BC in *Macrocystis* (Assis et al. 2023; Bemmels et al. 2026; Gierke et al. 2023), suggesting the existence of northern refugia where kelp persisted through the Last Glacial Period. Fine‐scale differences in genetic diversity are also observed, with lower diversity in the inner Salish Sea, fjords, and other inland waterways (Bemmels et al. 2025; Gierke et al. 2023), suggesting that these areas are more genetically isolated than the outer coast. In both species, turnover in genetic variation over local to regional scales has been attributed to factors such as habitat connectivity, ocean currents, geographic distance, and environmental differences (Alberto et al. 2010, 2011; Gierke et al. 2023; Johansson et al. 2015).

Although populations of wild species frequently differ across their genomes, an important management question is whether some of these differences reflect adaptation to local environments. If local adaptation exists, then ensuring a close genetic match to current or future environments could help managed populations thrive (Vranken et al. 2021). Local adaptation is traditionally assessed by reciprocal transplant studies that test whether local populations show higher fitness than foreign populations (Leimu and Fischer 2008). In *Nereocystis*, thermal tolerance is similar among different populations from Puget Sound, suggesting a lack of local adaptation (Fales et al. 2023; Weigel et al. 2023), though differences in thermal tolerance may yet be found if populations are sampled over a greater geographic distance (Weigel et al. 2023). In *Macrocystis*, a reciprocal transplant between a warm and cold site in BC found no consistent evidence of local adaptation, although the cold provenance consistently outperformed the warm provenance (Dykman et al. 2025), suggesting that genetic differences in adaptively relevant traits may exist between populations even if they do not conform to the expectations of local adaptation. Farther afield, Chilean *Macrocystis* populations were not found to exhibit local adaptation in terms of thermal tolerance (Becheler et al. 2022), yet other studies have identified local adaptation to temperature and pH among Chilean and Peruvian populations (Hollarsmith et al. 2020; Solas et al. 2024) and to temperature and nutrients in California and Mexico (Kopczak et al. 1991; Ladah and Zertuche‐González 2022). Overall, these studies suggest that variation in adaptive traits often exists among *Macrocystis* populations, though patterns of variation remain to be characterized in detail in the PNW.

As reciprocal field transplants are laborious, genotype‐environment association (GEA) analyses that identify correlations between allele frequencies and environmental variables offer a promising alternative method of inferring local adaptation (Lasky et al. 2023). This approach can identify environmental variables that drive genetic change (presumably due to local adaptation) and, conversely, predict how well‐adapted populations are to novel environments based on their genetic composition. In *Nereocystis*, a GEA analysis of populations from Puget Sound found evidence of adaptation to salinity, temperature, turbidity, and other variables (Abbott et al. 2025). Another GEA study of both species across BC and Washington inferred the presence of local environmental adaptation and predicted future genomic offsets (i.e., genetic maladaptation to environment) under different climate and management scenarios (Hernández et al. 2026). While GEA analyses are a powerful tool, their predictions have not been fully validated in kelp (Abbott et al. 2025; Hernández et al. 2026) and require testing in the field. However, correlations among predicted genomic offsets and observed kelp declines to date in wild populations (Starko et al. 2024) suggest that genomic offsets are indeed informative of overall extirpation risk (Hernández et al. 2026).

Although GEA analyses aim to identify differences in population allele frequencies driven by adaptation, allele‐frequency differences may also arise through genetic drift (i.e., random change over time due to chance), especially in small populations where the effects of genetic drift are more pronounced (Wright 1931). In BC and Washington, genomic estimates of population size vary by several orders of magnitude among *Nereocystis* populations but are more uniform in *Macrocystis* (Bemmels et al. 2025). Smaller populations exhibit reduced genetic diversity and increased inbreeding coefficients in both species, highlighting how small populations face multiple genetic risk factors (Bemmels et al. 2025). In particular, low genetic diversity may reduce the potential for a population to adapt to future conditions (Hoffmann et al. 2017). Meanwhile, increased inbreeding coefficients indicate that individuals are on average more closely related to one another in small populations than in large populations. Inbreeding often leads to inbreeding depression (i.e., the reduced fitness of inbred individuals) in wild species (Crnokrak and Roff 1999; Willi et al. 2022), which is primarily caused by rare recessive deleterious alleles that are more likely to be homozygous (allowing their negative fitness effects to be expressed) when an individual's parents are closely genetically related (Charlesworth and Willis 2009).

Self‐fertilization or selfing represents an extreme form of inbreeding and provides an opportunity to test for inbreeding depression in self‐compatible organisms. Due to short dispersal distances of most kelp spores, male and female gametophytes from the same parent often develop close enough together to successfully mate, such that selfing is expected to be common (Edwards 2022; Gaylord et al. 2006). Across BC and Washington, approximately 10% of adult sporophytes were inferred to be self‐fertilized in both *Nereocystis* and *Macrocystis* (Bemmels et al. 2025). In *Nereocystis*, selfing rates are negatively associated with population size, though selfing does occur in both large and small populations in both species (Bemmels et al. 2025). Negative fitness costs of selfing have been directly observed or predicted from spatial genetic models in *Macrocystis* from California (Johansson et al. 2013; Raimondi et al. 2004; San Miguel 2017). In Chile, tradeoffs between gametophyte fecundity and fertility suggest that selfing may result in similar overall fitness to outcrossing (Camus et al. 2021). However, the fitness effects of selfing have not been rigorously tested in either species in the PNW. Surprisingly, *Macrocystis* cultures from BC with higher rates of selfing exhibited higher survival than those with lower selfing rates in a field experiment (Dykman et al. 2025), which suggests that selfing may not have a large detrimental effect on fitness, but further study is warranted.

Although increased homozygosity of recessive deleterious alleles may reduce fitness of inbred individuals, a related phenomenon can occur at the population level. Especially in small populations (Wright 1931), strong genetic drift can cause random loss or fixation of mildly deleterious recessive alleles (Bertorelle et al. 2022). The alleles fixed this way are thus homozygous in all individuals of that population. Populations with a large number of such alleles are said to have high genetic load, which is predicted to cause reduced fitness (Bertorelle et al. 2022). Genetic load varies greatly among populations in BC and Washington and is negatively correlated with population size in *Nereocystis* but not *Macrocystis* (Bemmels et al. 2025). It is well known from crop breeding that the negative effects of high genetic load can sometimes be overcome by crossing different cultivars (Birchler et al. 2006; Paril et al. 2024). As different cultivars have each experienced independent genetic drift resulting in random fixation of different loci, crossing cultivars may result in hybrid vigor where heterozygosity is restored and offspring exhibit more desirable traits than either parent (Birchler et al. 2006; Paril et al. 2024). The conditions in which hybrid vigor is expected to occur have been predicted from genomic data in *Nereocystis* and *Macrocystis* (Bemmels et al. 2025) and field tests of hybrid and single‐population crosses are underway in *Nereocystis* from the north Salish Sea (L. Dykman et al., personal communication). In Chilean *Macrocystis*, evidence for hybrid vigor has been mixed: it has been variously observed in northern but not southern populations (Solas et al. 2024), stressful but not benign conditions (Murúa et al. 2021), or specific crosses only (Westermeier et al. 2010, 2011).

In summary, substantial population genetic knowledge has been assembled for *Nereocystis* and *Macrocystis* in the PNW from molecular genomic studies and lab and field experiments. We identify six key points:

1. Genetic variation is highly geographically structured within the PNW, likely as a result of limited migration and low dispersal between regions.
2. Genetic diversity is higher in northern areas of the PNW than in southern areas, and along the outer coast than in inner seas and fjords.
3. GEA analyses have used genomic data to infer local adaptation and predict the ideal genomic composition of local populations under future climates, but evidence of local adaptation from field trials is nuanced and requires further study.
4. Small populations face multiple risks including low genetic diversity, increased inbreeding rates, and high genetic load.
5. Self‐fertilization is common (~10% of adults in either species) and predicted to possibly reduce fitness based on theory, genomic data, and experimental evidence from California (*Macrocystis*), but experimental evidence of inbreeding depression is lacking in the PNW.
6. Crossing populations is predicted to result in hybrid vigor under certain circumstances, as has sometimes been observed in South American *Macrocystis*, but experimental evidence is lacking in the PNW.

In the following sections we highlight how these key points have important implications for the management of kelp populations through restoration, aquaculture, wild harvest, and biobanking.

## 3 Outplanting Kelp for Restoration and Aquaculture

Kelp restoration and aquaculture frequently involve the collection of genetic material from one or more wild populations, culturing genetic material in the lab to produce juveniles, and outplanting juveniles back into the wild at a location that may differ from the original collecting site. In all cases, the outplanted population should ideally thrive in its current environment and should not negatively impact nearby wild populations. In the case of restoration, the outplanted population should ideally persist over multiple generations and be able to adapt to future environments, while in the case of aquaculture, reproduction and long‐term persistence may sometimes be undesired. There are a variety of conceptual paradigms for kelp restoration that differ in the degree to which outplanted populations are intended to resemble historical genetic baselines. Coleman et al. (2020) delimited four paradigms: *recover* ecologically to an unknown genetic baseline; *revive* a known genetic baseline; *reinforce* the historical baseline with genetic improvements; and *redefine* the desired genetic baseline. We link our discussion below to these paradigms but do not advocate here for any particular approach; instead, we consider how existing population genetic knowledge can guide decisions about geographic transfer of genetic material, local source population selection, and genetic crosses.

### 3.1 Geographic Transfer of Genetic Material

Kelp restoration and aquaculture may involve transfer of genetic material from one geographic site to another. Geographic transfer could be required if there are no healthy native kelp populations available from which to collect near the outplanting site (Wood et al. 2020), if kelp from a different geographic area or a particular cultivar is believed to be genetically superior to local populations (e.g., due to higher genetic diversity, predicted genetic adaptation to current or future local environments, or possession of specific genetic composition known to result in desired phenotypes; Layton and Johnson 2021), or for practical reasons such as minimizing cost and labor. Current distance‐based guidelines regarding kelp transfer in the PNW remain fairly basic and informal (Cui 2023; Gruenthal and Habicht 2022; McConnell et al. 2024). There is great opportunity for kelp practitioners and policy makers to co‐develop more detailed guidelines that incorporate population genetic knowledge, a process which is in its early stages in jurisdictions such as BC (J. Schuster, personal communication) and Alaska (K. Gruenthal, personal communication).

A regulated approach to geographic transfer of kelp is warranted in the PNW as both *Nereocystis* and *Macrocystis* exhibit strong genetic structure (Figure 1) (Bemmels et al. 2025, 2026; Gierke et al. 2023) and genomic evidence of adaptation to environmental gradients (Abbott et al. 2025; Hernández et al. 2026). Given this situation, moving kelp over long distances is risky because outplanted kelp populations may not be well adapted to their new environments (Solas et al. 2024), leading to wasted restoration effort or failed aquacultural harvest. In addition, outplanted kelp may breed with local kelp populations (Hu et al. 2021), transferring maladaptive alleles to local populations or otherwise altering their genetic makeup. The offspring of introduced and local kelp could suffer from reduced fitness due to outbreeding depression (Edmands 2007; Lynch 1991), as has been observed among some populations of Chilean *Macrocystis* (Solas et al. 2024). The risk of harm is highest when local populations are extremely small and introduced individuals represent a significant proportion of the total kelp. In these cases, introduced individuals themselves may be insulated from natural selection due to restoration practices (i.e., culturing gametophytes in vitro), but the subsequent generations would be largely unfit hybrids of introduced and local individuals, potentially leading to immediate population collapse. Conversely, if introduced genetic variants are not maladaptive, high transfer of genetic material could lead to genetic swamping, whereby introduced genetic material eventually overwhelms a local population and leads to its replacement (Allendorf et al. 2001; Loureiro et al. 2015; Roberts et al. 2010). Genetic swamping is often viewed as undesirable in the context of species hybridization, where the loss of a local population represents the loss of an entire species and its unique genetic composition. The *integrifolia* and *pyrifera* morphs of *Macrocystis* remain genetically and morphologically distinct where they co‐occur in California (Gonzalez et al. 2023) despite the possibility of historical gene flow (Bemmels et al. 2026), suggesting that these morphs are resistant to gene swamping. However, even populations that are not morphologically distinct may have unique traits that are ecologically or culturally important. If local populations do not possess unique traits, genetic swamping may simply result in a genetically fitter kelp population and be of little immediate concern, although its effects are irreversible. Current approaches to prevent genetic swamping focus on requiring high genetic similarity between local and introduced populations, such that even if swamping were to occur the result would be little genetic change.

**FIGURE 2:**
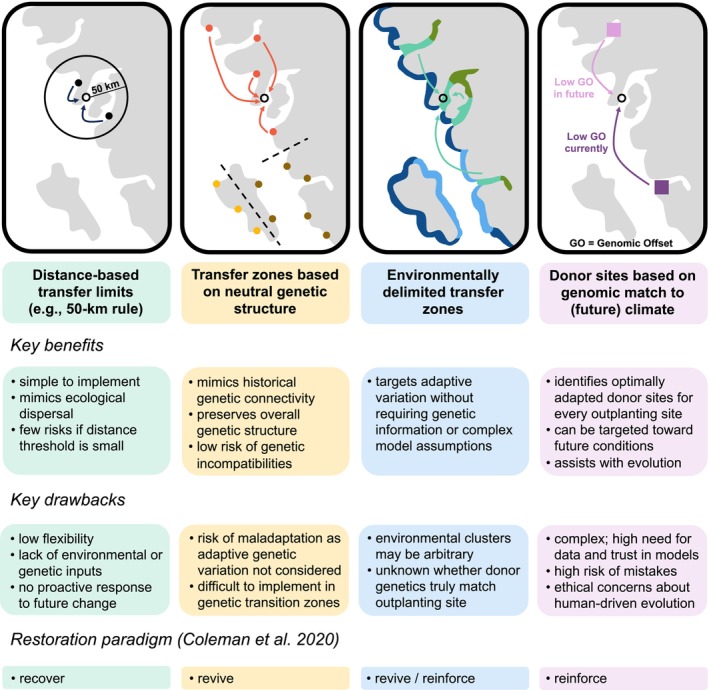
Potential approaches to transfer of kelp genetic material in the PNW, including key benefits and drawbacks and the restoration paradigm (Coleman et al. 2020) that most closely aligns with each approach. In each cartoon representation, grey and white correspond to land and water, respectively, the open black circle represents a target outplanting site for restoration or aquaculture, and arrows represent the movement of genetic material. Distance‐based transfer identifies potential donor sites (closed black circles) within a specific distance threshold (e.g., 50 km). For transfer zones based on neutral genetic structure, boundaries between zones (dashed lines) are based on neutral genetic clusters (closed circles of different colors). Environmentally delimited transfer zones permit transfer within regions of similar environmental conditions (solid colors). Donor sites based on the genomic match to climates use the predicted genomic offset (GO; see text for details) to identify donor sites (colored square grid cells) that are predicted to have genomic composition well suited to the environment of the target outplanting site in the present or future.

In part due to concerns about these perceived threats, the Alaska Department of Fish and Game implemented permitting guidelines in 2016 limiting kelp transfer to no more than 50 km by water from its site of origin (Gruenthal and Habicht 2022). This “50‐km rule” has subsequently been informally applied in British Columbia (McConnell et al. 2024) and Washington (Cui 2023). The “50‐km rule” was developed based on literature review of genetic structure and dispersal distance in multiple kelp species, but fine‐scale, species‐specific knowledge about genetic structure was not available at that time (Gruenthal and Habicht 2022). In the absence of such knowledge, the “50‐km rule” represents a scientifically sound, conservative approach that minimizes the risk of maladaptation, outbreeding depression, and genetic swamping, and corresponds closely to Coleman et al. (2020) *recover* paradigm of kelp restoration that uses ecological principles to avoid altering an unknown genetic baseline (Figure 2). However, as anticipated in the original formulation of the “50‐km rule” (Gruenthal and Habicht 2022), transfer > 50 km may be warranted in some situations. For example, a genetically similar population or one believed to be well adapted to a local environment may exist more than 50 km away. In contrast, the “50‐km” rule may be overly permissive in cases where there is rapid turnover in environmental gradients or genetic composition over fine spatial scales (such as observed in *Nereocystis* in the Broughton Archipelago; Bemmels et al. 2025).

An alternative to distance‐based guidelines could be the development of geographically or environmentally delimited transfer zones (Figure 2), similar to those used for managing terrestrial tree species in British Columbia since the 1970s (Ying and Yanchuk 2006). Transfer of genetic material would be permitted within but not between zones, in an approach that would align with Coleman et al. (2020) *revive* paradigm of kelp restoration that aims to replicate a known historical genetic baseline. Although there are multiple ways that such zones could be delimited, one of the most straightforward would be based on neutral population genetic structure, which has been characterized in detail in British Columbia and Washington (Bemmels et al. 2025; Gierke et al. 2023) and to a lesser extent in Alaska (Bemmels et al. 2026). Though formal zones were not delimited, neutral population genetic structure has informed restoration practice in Australia (Wood et al. 2020) and restoration guidelines in Scotland (Thomson 2021). The first‐order genetic clusters that exist within the PNW for *Nereocystis* and *Macrocystis* (Figure 1) could provide an initial basis for genetically delimited transfer zones, though delimiting zones in transition areas between clusters would be challenging. Transfer within zones based on genetic clusters would preserve the overall genetic relationships among different geographic regions that have evolved naturally over time, and would reduce the risks of both outbreeding depression (Frankham et al. 2011) and replacement of local populations through gene swamping, because introduced and local populations would be genetically similar. Alternatively, the application of the “50‐km rule” within broad transfer zones could further reduce these risks, highlighting how the different potential approaches to kelp transfer (Figure 2) are not mutually exclusive.

As neutral genetic variation does not impact fitness, transfer zones based on neutral genetic structure may not help outplanted kelp thrive in current or future environmental conditions. Especially in an environmentally complex coastline such as that of the PNW, nearby populations that resemble one another at neutral loci could inhabit very different environments. If populations are generally locally adapted, then using environmental information to inform transfer could help ensure that outplanted kelp possess adaptive genetic variation suited to their environments. One simple approach could be the development of environmentally delineated transfer zones (Figure 2). For comparison, in the BC forestry industry, the first formal seed transfer zones were based on environmental similarity under the assumption that trees from similar environments would be similar in terms of adaptive genetic variation (Ying and Yanchuk 2006). Deciding which environmental features would be most relevant for delimiting kelp genetic transfer zones and the ideal spatial scale of such zones (i.e., whether local or regional) would be difficult in the absence of detailed information on local adaptation. However, environmental clustering of variables selected based on expert opinion has been employed to analyze spatial trends in kelp forest persistence (Gendall et al. 2025; Mora‐Soto et al. 2024) and could represent a provisional approach to developing transfer zones that could be updated as evidence characterizing local adaptation accumulates from field trails.

As global change continues to alter environmental conditions in coastal marine ecosystems (Doney et al. 2012), genetic transfer guidelines will need to be updated to reflect future environmental conditions (O'Neill and Gómez‐Pineda 2021). GEA analyses provide a powerful, flexible, and predictive approach to guiding transfer of genetic material that can be adjusted to reflect changing environments. For example, GEA analyses and genetic diversity patterns have been used to qualitatively identify the Whidbey Basin as an ideal source region for genetic transfer to southern Puget Sound where *Nereocystis* is in decline (Abbott et al. 2025). More generally, the genomic offsets obtained from GEA analyses predict how well the putatively adaptive genetic variation from one site is expected to match the environment at another site or in another time period (Capblancq et al. 2020; Rellstab et al. 2021). Genetic transfer guidelines based on genomic offsets would not delineate specific zones, but would instead involve identifying donor sites where genomic composition is expected to closely match the ideal for an outplanting site (Figure 2), either now or in the future. Such an approach would closely align with the *reinforce* kelp restoration paradigm of Coleman et al. (2020) aimed at improving existing genetic baselines. Genomic offset predictions are available for both *Nereocystis* and *Macrocystis* throughout BC and Washington (Hernández et al. 2026). Promisingly, Hernández et al. (2026) demonstrated that for many geographic regions, using genomic offsets to guide kelp transfer results in markedly lower risk of a genetic mismatch under climate change relative to alternative strategies such as limiting transfer to 50 km or within genetic clusters. Despite the promise of these methods, GEA models and genomic offsets rely on a large number of assumptions that require validation before widespread application (Capblancq et al. 2020; Rellstab et al. 2021). In addition, actions that attempt to modify genetic baselines face complex ethical questions (Aubin et al. 2011; Coleman et al. 2020) that would require careful and informed consideration from stakeholders prior to their application.

### 3.2 Optimizing Sampling Site and Effort

Regardless of the framework used to guide kelp transfer (Figure 2), when there are multiple potential source populations for restoration or aquaculture that do not substantially differ in climate or other notable characteristics, we recommend collecting from the population believed to have the largest population size. Contemporary population size is positively correlated with genetic diversity in both *Nereocystis* and *Macrocystis* (Bemmels et al. 2025) and maintaining genetic diversity is a major conservation goal aimed at ensuring the long‐term adaptive potential of populations faced with unknown future challenges (Wernberg et al. 2018). However, the fitness benefits of higher genetic diversity may also extend to the present generation (Reed and Frankham 2003). The importance of diversity to performance remains to be broadly tested but was hinted at in a study of *Macrocystis* that found higher growth and survival of a genetically diverse population compared to a nearby genetically depauperate source population regardless of outplanting site (Dykman et al. 2025).

Once a source population has been identified, it is important to consider how many and which individuals to collect. The latter question is more straightforward to address: we recommend collecting from individuals as widely spaced apart as possible to avoid collecting close relatives or identical genotypes. As most kelp spores often disperse only a few metres from their parents (Edwards 2022; Gaylord et al. 2006; Reed 1990), individuals are expected to be more closely related to other individuals in their immediate vicinity than to those farther apart. This prediction was weakly supported in California *Macrocystis* where a weak but significant relationship was observed between pairwise kinship and distance over scales from 0.5 to 12 m (Johansson et al. 2013). First‐degree relatives (e.g., siblings, parent‐offspring pairs) were detected infrequently (*Nereocystis*: 4 of 491 individuals; *Macrocystis*: 5 of 260 individuals) from populations sampled across the PNW (Bemmels et al. 2026), suggesting that heuristic sampling approaches of requiring a minimum distance of a few metres between individuals were mostly effective at avoiding sampling close relatives. However, genetically identical pairs of individuals were detected more frequently (*Nereocystis*: 26 of 491 individuals; *Macrocystis*: 25 of 260 individuals). It is unclear whether this suggests the presence of vegetative reproduction, which has been reported in *Macrocystis* (Graham et al. 2007) but not to our knowledge in *Nereocystis*, or alternatively, that surface collectors across diverse collecting teams often mistake fronds of the same individual for those of different individuals. The latter scenario would imply that minimum collecting distance between individuals should ideally be much greater than only a few metres, though in small fringing kelp beds, collecting a large number of widely spaced individuals may not always be possible.

The question of how many individuals to collect is more difficult. The primary genetic considerations regarding population size are maintaining sufficient genetic diversity to ensure adaptive potential and avoiding inbreeding (Frankham et al. 2014). Current guidelines in Alaska require outplanted kelp stock to be derived from at least 50 unrelated wild individuals, based on heuristics from the conservation genetics literature balanced against practical considerations (Gruenthal and Habicht 2022). Empirical guidelines concerning the minimum number of individuals to conserve ex situ have frequently focused on allelic representation, or the proportion of alleles represented in a collection (Cibrian‐Jaramillo et al. 2013; Koontz et al. 2026; Lawrence et al. 1995; Wei and Jiang 2021), and could potentially provide a data‐informed decision‐making framework when collecting kelp stock destined for outplanting. Allelic representation targets are arbitrary (Koontz et al. 2026) but may include, for example, collecting enough individuals to represent 95% of all alleles or only alleles above a certain frequency in a population (Koontz et al. 2026; Lawrence et al. 1995).

It is well appreciated that allelic representation as a function of sample size can be predicted mathematically (Lawrence et al. 1995) and results in diminishing returns once all common alleles have been represented (Cibrian‐Jaramillo et al. 2013; Koontz et al. 2026). To illustrate these points empirically, we reanalyzed genomic data from Bemmels et al. (2025) for *Nereocystis* (sample sizes were not large enough for meaningful analysis in *Macrocystis*). We resampled different numbers of individuals from single nucleotide polymorphism (SNP) datasets thinned to a minimum distance of 10 kbp in the genome and subsetted to each of three geographic regions (Strait of Juan de Fuca: *n* = 56 individuals; Outer Coast of Vancouver Island and Washington: *n* = 108; Barkley Sound: *n* = 62) that each had ≥ 50 individuals available belonging to the same genetic cluster and with low genetic differentiation among populations (*F*_ST_ < 0.05 for all pairwise comparisons). For each number of resampled individuals, we randomly resampled individuals 100 times (or fewer if there were < 100 unique combinations of individuals) and recorded the number of alleles that were polymorphic (i.e., both SNP alleles were sampled). In addition to resampling empirical datasets, we also calculated the theoretical probability of sampling both alleles given different sample sizes and different minor allele frequencies using equation 1 of Edge et al. (2013).

**FIGURE 3:**
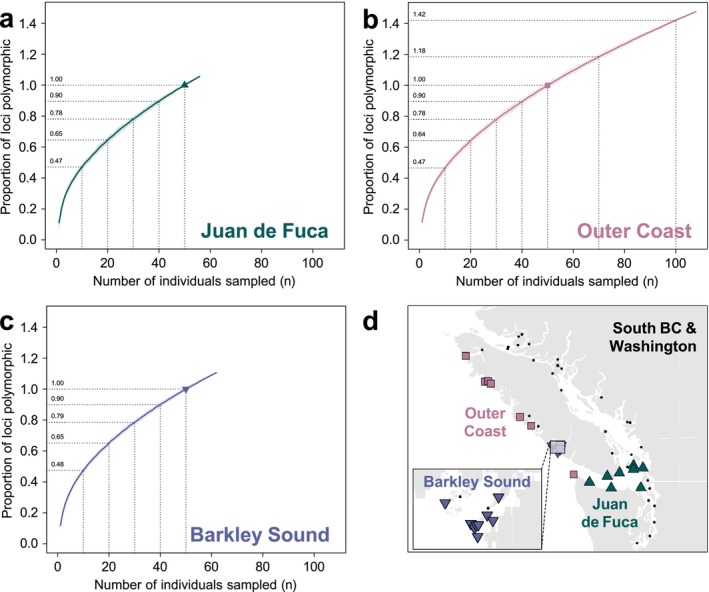
Allelic representation curves for *Nereocystis* based on resampling empirical genomic datasets. Resampling was performed for (a) the Strait of Juan de Fuca, (b) the Outer Coast of Vancouver Island and Washington, and (c) Barkley Sound. The proportion of SNP loci that are polymorphic (i.e., each of the two SNP alleles were sampled) is plotted as a function of the number of individuals sampled (*n*) and rescaled relative to a sample size of *n* = 50. Solid line: Mean of 100 resampling replicates; shaded polygon: Range containing 95% of resampled replicates. (d) Sampling sites included in each of the geographic regions, with colors and symbols as in (a–c). Small black dots represent additional sampling sites included in Southern BC and Washington in Figure 5. Note that Barkley Sound was analyzed separately (c) but is also included as part of the Outer Coast (b).

**FIGURE 4:**
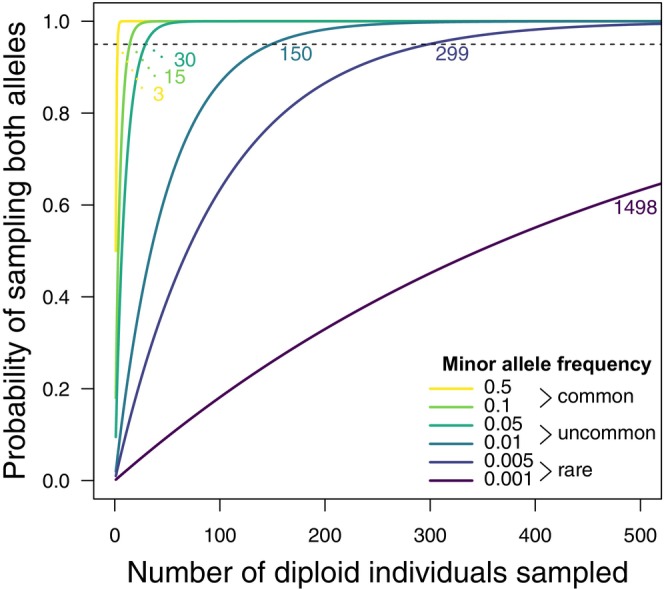
Theoretical probability of sampling both SNP alleles at a given locus when different numbers of individuals are sampled from a single population. Curves are plotted for SNPs of different minor allele frequencies, with the number of individuals required to attain a 95% probability (dashed line) indicated in coloured text.

For each of the three geographic regions and considering all alleles, allelic representation showed diminishing returns with increasing sample size but no sign of nearing its asymptote (Figure 3). Small sample sizes resulted in relatively high allelic representation: only 10 individuals could represent almost half (0.47–0.48) of the genetic variation that would have been obtained relative to sampling 50 individuals, and even a single individual resulted in > 10% of this value. In contrast, increasing sample size from 50 to 100 individuals resulted in only a 42% relative gain in allelic representation (Figure 3b). These results are consistent with theoretical expectations (Figure 4) that very few individuals need to be collected in order to sample both alleles when the minor allele is common, but large sample sizes are needed when the minor allele is rare. For example, a sample size of 30 is sufficient to reach a 95% probability of sampling both alleles at a minor allele frequency of 0.05, whereas the same probability at a minor allele frequency of 0.001 would require nearly 1500 individuals. Increasing the sample size from 30 to 50 individuals would increase the probability of sampling both alleles from 95% to > 99% at minor allele frequency (MAF) 0.05, from 45% to 63% at MAF 0.01, and from 6% to 10% at MAF 0.001. While these empirical and theoretical trends do not provide a definitive answer to the question of how many wild individuals to collect, they do provide kelp managers with quantitative predictions about the loss of genetic diversity (relative to wild source populations) that is expected in outplanted stock derived from different numbers of parents, and reinforce the intuition that operationally realistic sample sizes (i.e., dozens of unrelated parents; Gruenthal and Habicht 2022) are likely to do an excellent job at representing almost all of the common genetic variation in a kelp population, whereas very large sample sizes would be necessary to avoid losing rare variants.

In addition to minimizing loss of genetic diversity, the number of individuals used to generate outplanted stock has bearing on the rate of inbreeding, which may occur due to selfing in lab cultures or among the closely‐related offspring of outplanted individuals in subsequent generations. Whether inbred kelp experience inbreeding depression has not been rigorously tested in most PNW populations (cf. Dykman et al. 2025), but due to evidence of inbreeding depression in California *Macrocystis* (Johansson et al. 2013; Raimondi et al. 2004; San Miguel 2017) and a strong theoretical possibility of inbreeding depression in wild species in general (Crnokrak and Roff 1999; Willi et al. 2022), practitioners may wish to avoid inbreeding unless future evidence emerges that it is not a cause for concern. The predicted rate of selfing in a gametophyte culture produced from multiple parents with random mating and equal reproductive success of all parents is proportional to the inverse of the number of parents, such that inbreeding can be predictably reduced by increasing the number of parent individuals. Among *Nereocystis* and *Macrocystis* wild adult sporophytes from British Columbia and Washington, the average rate of selfing detected from genomic data is approximately 10% in both species (Bemmels et al. 2025). Thus, only 10 unrelated parent individuals (1/10 = 0.1) are needed to replicate the average selfing rate in the wild. Increasing to 50 parent individuals would result in very low selfing rates (1/50 = 0.02), suggesting that outplanted kelp following current guidelines from Alaska (Gruenthal and Habicht 2022) may experience lower rates of selfing than many wild populations. Observed selfing rates in *Macrocystis* cultures produced from different numbers of parents (Dykman et al. 2025) matched these expectations well (two parents: expected rate 0.5, observed rate 0.5 from *n* = 30 genotyped offspring; 10 parents: expected rate 0.1, observed rate 0.1875 from *n* = 16 offspring), suggesting that selfing readily occurs at close‐to‐expected rates in *Macrocystis* cultures. Thus, increasing the number of parent individuals may be an effective means of decreasing inbreeding in kelp cultures. However, further study is warranted, especially given that in *Ecklonia* a dramatic increase in inbreeding indicative of unequal reproductive success has been observed in mass‐spawned hatchery cohorts of 100 parents, suggesting that a large number of parents may not be sufficient to prevent inbreeding under all kelp culturing conditions (Wood et al. 2026).

As a caveat, we note that just as inbreeding rates can vary among populations, the strength of inbreeding depression can also vary. Interestingly, Bemmels et al. (2025) predicted that small populations of *Nereocystis* are likely less susceptible to inbreeding depression than large populations. The intuition to explain this counterintuitive prediction is that in smaller populations, genetic drift has caused fixation of higher numbers of the recessive deleterious alleles that contribute to inbreeding depression when in a homozygous state (Charlesworth and Willis 2009). When alleles are already fixed in a small population, mating with a close relative does not increase the probability that these fixed alleles will be homozygous in the offspring compared to mating with an unrelated individual (in other words, inbred and outbred individuals alike both suffer negative fitness consequences of these alleles). This prediction has not been tested in the field, but if it holds true, it suggests that avoiding inbreeding depression is less of a concern for small than large populations. Thus, if kelp managers are unable to obtain reproductive tissue from a large number of individuals from an endangered population that has historically been very small, concerns about inbreeding depression should not prevent outplanting of a culture produced from a smaller number of parents (Bemmels et al. 2025). However, we always recommend using more parent individuals if available due to the increase in genetic diversity that more parents would provide. Furthermore, a historically small population that has experienced substantial genetic drift may have lower fitness overall than a historically larger population, so the possibility that inbreeding is relatively less harmful within the small population than within the large populations does not imply that small source populations should be preferred when selecting among alternative donor populations.

### 3.3 Genetic Crosses

Thus far we have considered the outplanting of kelp derived from a single source population, but the crossing of populations should also be considered as a means of replicating the genetic structure and diversity of historical populations that have been lost (Wood et al. 2020) or potentially boosting performance of outplanted individuals through hybrid vigour. Crossing cultivars to produce hybrid vigour has been a key feature of kelp breeding success in Asia (Goecke et al. 2020) and experimentally tested in North Atlantic *Laminaria* (Liesner et al. 2022). Further research is needed to confirm whether and in what conditions hybrid vigour might occur in *Nereocystis* and *Macrocystis* in the PNW, especially given the mixed evidence of hybrid vigour in *Macrocystis* population crosses in Chile (Murúa et al. 2021; Solas et al. 2024; Westermeier et al. 2010, 2011). However, Bemmels et al. (2025) used genomic data to examine genetic load and predict the relative strength of hybrid vigour in different simulated genetic crosses. They predicted that hybrid vigour (resulting from a reduction in genetic load through the restoration of heterozygosity at recessive deleterious alleles) would be strongest in crosses between geographically distant populations and when the recipient population is small (Bemmels et al. 2025). These predictions suggest that introducing foreign genetic material through crosses could be especially useful in restoration of small, geographically isolated populations, where an influx of new genetic material could also increase genetic diversity and boost adaptive potential.

Crossing distant populations as a kelp management tool would require extensive testing to ensure desired outcomes, and careful ethical consideration is needed for actions that alter the genetic baseline of local populations (Coleman et al. 2020). In addition, hybrid vigour is expected to severely decline after the first generation, as heterozygosity declines (Charlesworth and Willis 2009; Edmands 2007; Lynch 1991). Nonetheless, single‐generation hybrid vigour could still be useful in restoration for helping a new population become initially established, or in aquaculture where a new population is outplanted and harvested every year. In addition, it is important to note that although genetic data suggest migration between distant populations is rare, rafting kelp can move for hundreds to thousands of kilometers (Bernardes Batista et al. 2018; Layton et al. 2022; Selivanova and Zhigadlova 1997). Infrequent rafting events are likely not sufficient to homogenize populations, but could be important for spreading genetic variation across the species. This means that the long‐distance movement of populations to create crosses for restoration and aquaculture would not be unprecedented on an evolutionary timescale.

### 3.4 Aquaculture‐Specific Considerations

Genetic interbreeding between cultivated and wild populations has been frequently observed in China and Japan (Hu et al. 2021) and remains a significant concern for the kelp farming industry in Europe and the Americas (Grebe et al. 2019). Because of the possibility that interbreeding with farmed populations could alter the genetic composition of nearby wild populations, we consider the above considerations about the genetic composition of outplanted kelp stock to be applicable in both restoration and aquaculture contexts. However, as aquaculture may employ specific kelp genotypes or populations that perform well for traits of commercial interest (Goecke et al. 2020; Hu et al. 2024; Hwang et al. 2019; Westermeier et al. 2010), it may be undesirable for these genotypes to genetically interact with nearby populations (Grebe et al. 2019; Hu et al. 2021). Fears also exist that high‐performing commercial cultivars might outcompete nearby wild populations (Vissers et al. 2024). To mitigate these concerns, kelp farms could be located far from wild kelp beds to minimize the risk of interaction, though areas without any wild kelp may be unsuitable for aquaculture. Alternatively, as pneumatocysts can be readily removed from *Nereocystis* resulting in the death of the individual kelp (Springer et al. 2007), commercial harvest could be timed prior to spore release with complete pneumatocyst removal to prevent reproduction and interbreeding with wild populations. Such a strategy would be ineffective in *Macrocystis* attached to the sea floor, where typical harvesting techniques do not kill the individual (Springer et al. 2007), but if attached to a removable substrate such as a seeded line then the entire individual could be removed. Active interest in techniques to produce sterile kelp cultivars may provide a future means of eliminating concerns about the escape of genetic material from kelp farms (Grebe et al. 2019; Vissers et al. 2024).

## 4 Wild Harvest

The harvesting of wild kelp has been practiced for millennia by coastal peoples and is now performed at an industrial scale in many regions of the world (Mac Monagail et al. 2017). Although harvest of wild kelp is not permitted in Washington except in small quantities for personal and traditional uses (WSL 2005), commercial harvest is allowed and regulated in British Columbia (Government of BC 2025) and Alaska (Ulaski et al. 2020). Sustainable wild kelp harvest involves numerous ecological and social considerations, which have been reviewed elsewhere (Mac Monagail et al. 2017). However, population genetic considerations can inform wild kelp harvest by helping minimize genetic risks to wild populations (Allendorf et al. 2008). As genetic changes may result when individuals are removed from a population or their reproductive output is altered, such changes may be especially relevant to consider for *Nereocystis*, given that stand‐level biomass recovery after harvest may take several months and can sometimes result in death of the harvested individual even when the pneumatocyst is left intact (Krumhansl et al. 2017; Ulaski et al. 2020). As pneumatocyst and stipe removal invariably results in death, it is prohibited during wild harvest of *Nereocystis* in British Columbia and Washington (Springer et al. 2007) but is believed to commonly occur in Alaska where the stipes are used for food products (Garza 2005; Ulaski et al. 2020). In contrast, *Macrocystis* harvest often involves pruning of only the non‐reproductive (Graham et al. 2007) top portion of the individual and is non‐lethal (Springer et al. 2007; van Tamelen and Woodby 2001). Rapid biomass recovery after small‐scale harvest of *Macrocystis* using these techniques (Krumhansl et al. 2017; van Tamelen and Woodby 2001) suggests that the potential for substantial genetic changes to harvested populations is minimal. However, more invasive harvest methods have been associated with both decreased reproductive output (Geange 2014; Reed 1987) and increased recruitment (Westermeier et al. 2014), suggesting that the capacity for wild harvest to genetically impact *Macrocystis* should not be discounted.

Allendorf et al. (2008) highlight three major ways in which wild harvest genetically impacts populations. Firstly, wild harvest can cause a loss of genetic variation by decreasing population size and increasing the effects of genetic drift (Allendorf et al. 2008). In kelp, these impacts could be minimized by using non‐lethal harvest techniques that allow individuals to continue growing and complete their life cycles (Krumhansl et al. 2017; Springer et al. 2007; van Tamelen and Woodby 2001). If lethal harvesting methods are employed, then allelic representation curves (Figure 3) can guide expectations about how much genetic diversity is expected to be lost when population sizes are reduced to a given number of reproductive individuals (assuming no migration from neighbouring patches). Secondly, harvest can cause changes in genetic structure (i.e., genetic subdivisions among populations) by reducing local densities and thus altering rates of migration among populations and recruitment from local vs. non‐local sources (Allendorf et al. 2008). In kelp, changes to genetic structure could be minimized by harvesting from individuals in a spatially dispersed fashion but leaving intervening individuals intact and able to genetically contribute to the next generation. In contrast, harvesting of an entire kelp patch in a clear‐cut fashion could result in recolonization from a distant source that might have a different genetic composition or not be as well adapted to local conditions. Timing harvest to occur after peak spore production, especially in the annual species *Nereocystis*, could also ensure that existing individuals are able to contribute to the next generation. Finally, wild harvest can cause selection for specific traits (Allendorf et al. 2008). In *Macrocystis*, since individuals impacted by biomass removal may show reduced reproductive output (Geange 2014; Reed 1987), harvesting from the surface (Krumhansl et al. 2017; van Tamelen and Woodby 2001) could potentially cause selection for slower growth rate, as smaller individuals at the time of collecting would be unaffected. In *Nereocystis*, harvesters intending to make food products typically prefer to collect clean blades free of epiphytes (Ulaski et al. 2020), which could potentially cause selection for lower epiphyte resistance and higher disease susceptibility as biofouled individuals would be left unharvested. Whether these types of harvester‐induced selection would be consistent and strong enough to effect meaningful genetic change is unknown, but the risk thereof could be minimized by collecting in a randomized fashion that targets individuals regardless of size or phenotype.

Carefully selecting a geographic site for wild harvest can support ecological sustainability (Mac Monagail et al. 2017) and minimize susceptibility to the above‐mentioned negative genetic effects. Genetic concerns do not fundamentally alter, but instead reinforce ecological considerations: harvesting from small, isolated, and genetically depauperate populations should be avoided when alternatives exist, as these populations may be the least likely to quickly ecologically recover and the most likely to experience strong genetic drift (Wright 1931) and loss of genetic diversity when population size is further reduced. In general, large, genetically diverse populations that are ideal candidates for wild harvest tend to occur in Alaska, northern British Columbia, and more ocean‐adjacent areas of the outer coast of Vancouver Island in both *Nereocystis* and *Macrocystis* (Bemmels et al. 2025, 2026). In contrast, smaller and lower‐diversity populations tend to occur in inner areas of fjords and other waterways, including in the Salish Sea (Bemmels et al. 2025, 2026); we consider these regions to be less appropriate candidates for large‐scale commercial wild harvest. However, our recommendations should not be construed as discouraging small‐scale wild harvest using less invasive harvesting techniques (Krumhansl et al. 2017; Springer et al. 2007; van Tamelen and Woodby 2001) informed by traditional ecological knowledge or monitoring.

## 5 Biobanking

Ex situ germplasm repositories (i.e., biobanks) are collections that can preserve genetic material to support research, conservation, and industrial needs (Day and Stacey 2008). Kelp biobanks involve preservation of live haploid gametophytes under dormancy conditions or cryopreservation (Barrento et al. 2016; Coleman et al. 2025; Schenk et al. 2026; Wade et al. 2020). Although several biobanks exist in Asia for the purpose of maintaining known cultivars of commercial species (Wade et al. 2020), biobanking efforts in other regions of the world have largely suffered from a lack of international coordination and systematic design (Hoffmann et al. 2017; Schenk et al. 2026; Wade et al. 2020). There is great potential for population genomics to support the design and evaluation of emerging biobanking initiatives, including decisions about what material to biobank and monitoring for unintended genetic changes in biobanked material.

As biobanks can have multiple goals (Day and Stacey 2008; Wade et al. 2020), decisions about which populations or cultivars to represent will heavily depend on the intended purpose of a biobank. If ex situ replication of wild biodiversity is a desired goal, then knowledge of population genetic structure (Figure 1) can be used to ensure that a biobank includes adequate specimens from all desired genetic clusters. Similarly, geographic patterns of genetic diversity can guide collection from the highest‐diversity populations (such as northern British Columbia, Alaska, and outer Vancouver Island; Bemmels et al. 2025, 2026), as targeting collection from such populations could result in the maximum retention of genetic diversity with minimal sample size. Sampling from high‐diversity regions that served as glacial refugia (i.e., north‐central British Columbia; Bemmels et al. 2026) could also be prioritized, as these populations may contain unique genetic diversity that was lost from more recently colonized areas during bottlenecks associated with postglacial range expansion. However, if protecting against the loss of unique genotypes is a more urgent goal than representing overall genetic diversity, then practitioners may wish to target small, genetically depauperate, and highly genetically differentiated populations that may be at high risk of extirpation in the wild. GEA studies could also be used to ensure representation of putatively adaptive genetic variation, by identifying environmental clusters predicted to have similar allele frequencies of adaptive variation (Abbott et al. 2025; Hernández et al. 2026). Practitioners could then use these clusters in a manner analogous to neutral genetic clusters, to ensure overall representativeness of differently adapted groups in the biobank and to prioritize representation of adaptive outlier populations that could contain unique adaptive genotypes.

**FIGURE 5:**
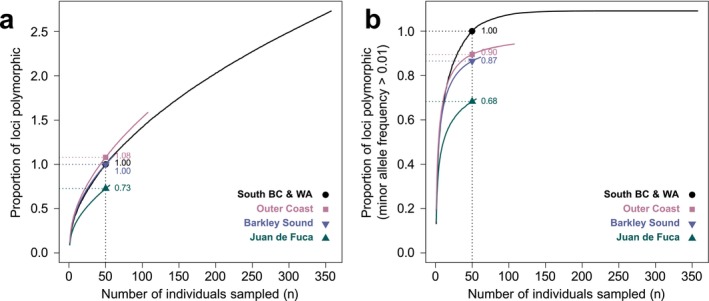
Allelic representation curves for *Nereocystis* based on resampling empirical genomic datasets under different geographic sampling strategies for (a) all loci and (b) only common loci with a minor allele frequency > 0.01. The three regions Outer Coast, Barkley Sound, and Juan de Fuca are subsets of the broader region of Southern BC and Washington (Figure 3d). The proportion of SNP loci that are polymorphic (i.e., each of the two SNP alleles were sampled) is plotted as a function of the numbers of individuals sampled (*n*) and rescaled relative to a sample size of *n* = 50 for Southern BC and Washington. For example, the pink text 1.08 in panel (a) indicates that sampling 50 individuals from only the Outer Coast results in an 8% relative increase in polymorphic loci relative to sampling 50 individuals broadly from across Southern BC and Washington. Solid lines represent the means of 100 resampling replicates.

Allelic representation curves (Figure 3) are a useful tool for determining how many individuals to preserve to balance maximal retention of biodiversity against available resources. However, biobanking a large number of individuals per population from multiple populations would quickly result in redundant allelic representation if populations share genetic variation. To explore this issue, we repeated our calculation of allelic representation curves for *Nereocystis* (as previously described) using all variant sites and again using only common variants with a frequency > 1%. To simulate a strategy of broad geographic sampling, we first sampled a single individual from each population (in random order) before sampling a second individual from an already‐sampled population, and so forth. We conducted 100 sampling replicates for each number of individuals and used datasets filtered to include all populations from southern British Columbia and Washington (Figure 3d), as well as only populations from each of the three geographic regions previously described. When there was no minor allele frequency cutoff, allelic representation curves showed no sign of nearing an asymptote (Figure 5a). Surprisingly, for the same number of individuals sampled (*n* = 50), sampling broadly across all of BC and Washington resulted in intermediate allelic representation compared to sampling only the high‐diversity Outer Coast region (8% higher allelic representation), Barkley Sound (equivalent representation), or the comparatively lower‐diversity Strait of Juan de Fuca (27% lower). However, when considering only common genetic variants, allelic representation approached an asymptote and sampling across all of BC and Washington resulted in higher allelic representation than sampling within a geographic subregion (Figure 5b).

Together, these results suggest that sampling from high‐diversity geographic regions may capture more alleles total than spreading out sampling broadly among high‐ and low‐diversity regions (Figure 5a), likely because high‐diversity populations harbor numerous rare alleles that continue to be newly captured with very large sample sizes. However, geographically broad sampling captures a higher proportion of common alleles (Figure 5b), likely because some globally common alleles are not present (or not common) in specific geographic regions. Thus, an ideal biobanking strategy for representing overall genetic diversity may be broadly sampling small numbers of individuals from numerous widely geographically spaced populations, combined with targeted sampling of large numbers of individuals from the most genetically diverse populations. One caveat to this recommendation is that overall allelic representation in a biobank may not adequately reflect all aspects of genetic diversity worth conserving, as different populations may possess different combinations of alleles that jointly impact phenotypes. Sampling across multiple populations, including across low‐diversity populations even if it results in increased allelic redundancy, would likely preserve a larger number of unique allelic combinations, though our simple allelic representation curves are unable to quantify this trade‐off.

Once genetic diversity is housed in a biobank, care must be taken to prevent genetic erosion, or the loss of genetic diversity over time. Genetic erosion in biobanks can occur due to unintentional selection or genetic drift (Wei and Jiang 2021). The environment of the biobank, regardless of whether individuals are kept under dormancy conditions or cryopreserved (Wade et al. 2020), could exert selective pressure if certain genotypes are more likely to survive preservation. Even without selection on genotypes, genetic drift is expected to have strong effects in any small population (Wright 1931), including those preserved ex situ (Wei and Jiang 2021), due to chance differences in reproductive success or growth rates of different individuals. For example, if populations are represented by mixed cultures where spores are released from multiple parents, different parents may happen to produce unequal numbers of gametophytes at the initial establishment of the culture. Once gametophytes are established and growing in culture (prior to dormancy or once revived), further drift could occur if different individuals happen to experience different growth rates over time, resulting in unequal biomass accumulation over time. When gametophytes reproduce to produce sporophytes for restoration or aquaculture, unequal reproductive output among gametophytes could result in further genetic drift affecting the outplanted population. Concerns about selection and drift could be minimized by maintaining separate cultures each derived from a single diploid parent or from a single haploid spore, though a large number of such cultures would be much more resource‐intensive to maintain than a smaller number of mixed cultures. Maintaining multiple replicates of the same mixed culture would also reduce the overall loss of diversity due to drift (but not selection), as the effects of drift would be different and random in each replicate. As little is known about the extent to which genetic erosion occurs in kelp biobanks (though research is underway; R. Nagel et al., personal communication), we recommend monitoring for genetic erosion by comparing genetic diversity of wild, ex situ, and outplanted kelp populations as a biobanking best practice.

## 6 Conclusions and Future Directions

Although studies of *Nereocystis* and *Macrocystis* have described population genetic structure and genetic health (Assis et al. 2023; Bemmels et al. 2025, 2026; Gierke et al. 2023) and generated predictions about strategies for minimizing inbreeding depression, leveraging hybrid vigour, and ensuring future environmental adaptation (Abbott et al. 2025; Bemmels et al. 2025; Hernández et al. 2026), most of these predictions remain untested. Field trials are sorely needed in the PNW (but see Dykman et al. 2025; Weigel et al. 2023). To address this knowledge gap, we suggest that field ecologists, restoration biologists, and aquaculture specialists routinely incorporate population genetic considerations about geographic provenance, genetic diversity, inbreeding depression, and hybrid vigour into their research activities in a controlled, experimental manner. As kelp are routinely collected from the wild and crossed in lab cultures prior to outplanting, such experiments could easily be incorporated into existing methodological pipelines. These experiments would provide valuable validation (or rejection) of the predictions discussed in this paper, and could ultimately lead to more successful restoration and aquaculture.

There are numerous additional potential applications of genetic knowledge to support kelp conservation and aquaculture, but many of these are outside the scope of population genomics per se or are poorly developed for kelp in the PNW. Selective breeding programs could be employed to develop phenotypically desirable and stress‐resistant kelp cultivars, following the example of decades of successful breeding in East Asia (Hu et al. 2021, 2024; Hwang et al. 2019) and preliminary efforts in *Macrocystis* in Chile (Camus et al. 2018; Westermeier et al. 2010, 2011) and Australia (Iha, Layton, Amancio, et al. 2023; Layton et al. 2020). Beyond traditional breeding methods, more advanced synthetic biology approaches (including gene editing) could potentially be employed in the future to engineer novel genetic variation in accordance with the *redefine* paradigm of kelp restoration of Coleman et al. (2020). However, enormous knowledge gaps (Hu et al. 2024) and ethical challenges (Coleman et al. 2020; Coleman and Goold 2019) associated with synthetic biology have prevented it from being more widely applied to kelp research to date. Of more immediate interest, thermal priming—the act of exposing individuals to altered temperature regimes, with the aim of conferring greater resilience to high temperatures later in life or possibly in the subsequent generation (Jueterbock et al. 2021; Wang et al. 2017)—could represent an alternative method of producing more desirable kelp stock without altering the underlying DNA nucleotide sequence itself. Thermal priming is being actively researched in *Macrocystis* and *Nereocystis* (Hotz et al. 2025; L. Coleman, personal communication). Although such research primarily falls into the realm of epigenetics, population genomic perspectives may still be useful if individuals or populations are found to differ in their amenability to thermal priming, in which case “primability” may behave like any other phenotypic trait subject to selection, genetic drift, and other evolutionary forces.

Overall, population genomic knowledge of canopy‐forming kelps has dramatically increased in the PNW in recent years. Genomic studies have allowed detailed characterization of genetic structure, genetic health indices, and patterns of environmental adaptation, and facilitated specific predictions about strategies that could be used to optimize performance of outplanted kelp in restoration and aquaculture, protect genetic integrity of populations subject to wild harvest, and inform biobanking efforts, though validating predictions derived from genomic analyses in a lab or field setting remains an important initial step. We believe that opportunity is ripe for ecologists, restoration biologists, kelp farmers, policy makers, Indigenous stewards, and other stakeholders to incorporate recent insights from population genomic studies into their existing practice. Ultimately, an understanding of genomic baselines, experimental validation of population genomic predictions, and careful stakeholder consideration of competing conceptual paradigms for managing kelp genetic diversity (Coleman et al. 2020) should all be incorporated into decision‐making and policy development to support kelp conservation and management. These considerations will be especially important as growth of the emerging seaweed industry in the PNW (Kim et al. 2019; Martone et al. 2025) places greater human pressure on kelp habitats and climate change continues to put many kelp populations at risk (Mora‐Soto et al. 2024; Starko et al. 2024).

## Conflicts of Interest

The authors declare no conflicts of interest.

## Data Availability

Raw genome sequencing data were previously published and are available at NCBI (NCBI SRA: PRJNA1164249; only *Nereocystis* samples not subject to a Biocultural Notice were reanalyzed in this study).
